# Conceptual frameworks and future directions for functional cognitive disorders in adults: a narrative review and integrative perspective

**DOI:** 10.1097/YCO.0000000000001062

**Published:** 2026-01-08

**Authors:** Adith Mohan, Catherine J. Hayes, Arun V. Krishnan

**Affiliations:** a Neuropsychiatric Institute, Prince of Wales Hospital, South Eastern Sydney Local Health District (SESLHD); b Centre for Healthy Brain Ageing (CHeBA), Discipline of Psychiatry and Mental Health, School of Clinical Medicine, Faculty of Medicine and Health; c School of Clinical Medicine, Faculty of Medicine and Health, UNSW Sydney; d Department of Neurology, Prince of Wales Hospital, South Eastern Sydney Local Health District (SESLHD), Sydney, Australia

**Keywords:** functional cognitive disorder, functional neurological disorder, internal inconsistency, metacognition, predictive processing

## Abstract

**Purpose of this review:**

This narrative review provides an overview of functional cognitive disorder (FCD) as a cognitive subtype within the functional neurological disorder (FND) spectrum. It addresses the conceptual challenges, diagnostic criteria, and epidemiology of FCD, emphasizing the need for standardization of internal inconsistency and clearer diagnostic boundaries to improve clinical assessment and research.

**Recent findings:**

FCD is characterized by persistent cognitive complaints disproportionate to objective performance, underpinned by metacognitive, attentional, and cognitive-behavioural dysfunction. Emerging evidence supports a predictive processing framework in which maladaptive top-down priors and attentional dysregulation perpetuate subjective cognitive deficits despite preserved or inconsistent objective cognitive performance. Diagnostic criteria and FCD checklists show promise, although challenges remain in standardizing neuropsychological assessments and integrating patient-reported experiences. Epidemiological data highlight the stability of FCD and its distinctiveness from neurodegenerative conditions, with a nonprogressive trajectory in most cases.

**Summary statement:**

Defining and refining FCD through standardized criteria and mechanistic models is crucial for enhancing diagnostic accuracy, patient care, and research validity. Advancing our understanding of the pathophysiology of FCD within the FND framework will facilitate targeted interventions and improve trial cohort purity in neurodegenerative disease research. Future studies should focus on objective biomarkers and therapeutic strategies that address attentional and metacognitive dysfunction in FCD.

## INTRODUCTION

Functional neurological disorder (FND) is a common disabling neuropsychiatric condition presenting with motor, sensory, seizure-like and cognitive phenomena, which often co-exist [1]. Historically stigmatized and misunderstood, FND is now understood as a multinetwork disorder involving disrupted information processing in key neural networks underpinning agency, attention, emotion processing, interoception, and predictive processing [2]. Diagnosis is made on an inclusionary rather than a ‘rule-out’ basis, with internal inconsistency and symptom variability representing hallmarks of positive clinical signs required for FND diagnosis. The modern framework of FND recognizes psychological factors (stress, trauma, developmental adversity) while allowing diagnosis even when such factors are absent or unable to be elicited. The patient experience is legitimized as involuntary as is the significant distress and disability caused by the condition.

A defining feature of FND is variability in primary presenting symptoms across patients. The standardization of the term “Functional”, while not without its critics [3], has allowed presentation rates to be better quantified. Overall, FND has an estimated incidence of 10–22 per 100 000 population per year and a minimum prevalence of 80–140 per 100 000, though estimates of prevalence range from 50–1600 per 100 000 due to methodological heterogeneity across studies [4^▪▪^]. While FND encompasses various motor and sensory subtypes [5], this review focuses on the significant prevalence of cognitive complaints within FND [5], which impact disability [6], and present unique challenges for clinical assessment and research. Cognitive symptoms affect between 40 and 70% of patients [7^▪^], overlap across FND subtypes [8] and significantly impact health-related quality of life, highlighting the need to understand and manage cognitive phenomena early in illness course [9].

Cognitive complaints, including clouded thinking, inattention, and memory difficulties, are reported in most people with FND [10], often alongside sensorimotor manifestations interfering with work, study and daily life. Historically, the nosological status of these cognitive symptoms was uncertain with dyscognition often excluded from FND definitions in favour of motor and sensory phenotypes [11]. Literature examining objective neurocognitive performance in FND shows heterogeneity in observable and reported deficits, presenting a diagnostic challenge [12]. This mismatch between subjective complaints and inconsistent objective performance highlights a significant conceptual challenge.

More recently, functional cognitive disorder (FCD) has emerged as an evolving diagnostic entity, conceptualized as the cognitive subtype within the FND umbrella. FCD is characterized by persistent cognitive difficulties in attention, concentration, and memory, where these symptoms are the primary driver of impairment. Crucially, its diagnosis relies on positive features of internal inconsistency and the absence of a better explanation by other conditions [13]. Relative to the heterogeneous, transdiagnostic syndrome of “brain fog”, FCD offers a narrower operationalized diagnosis aligned with the positive, nonexclusionary approach in FND. While “brain fog” can be associated with conditions ranging from anxiety and depression to sleep apnoea, chronic pain, fibromyalgia, and postconcussion syndrome [14], FCD is hypothesized to involve specific psychological and metacognitive factors, triggered by acute precipitants of pathophysiological significance [15^▪▪^].

We provide a narrative overview of FCD as a subtype of FND and its significance in advancing the clinical research in FND and other neurocognitive disorders alike. We briefly describe mechanistic evidence underlying FCD and its alignment with emerging literature on FND pathophysiology, acknowledging gaps in operationalizing FCD as a diagnostic subtype. Although therapies for FCD are at various stages of development, we have opted to leave these out of this paper's scope. 

**Box 1:**
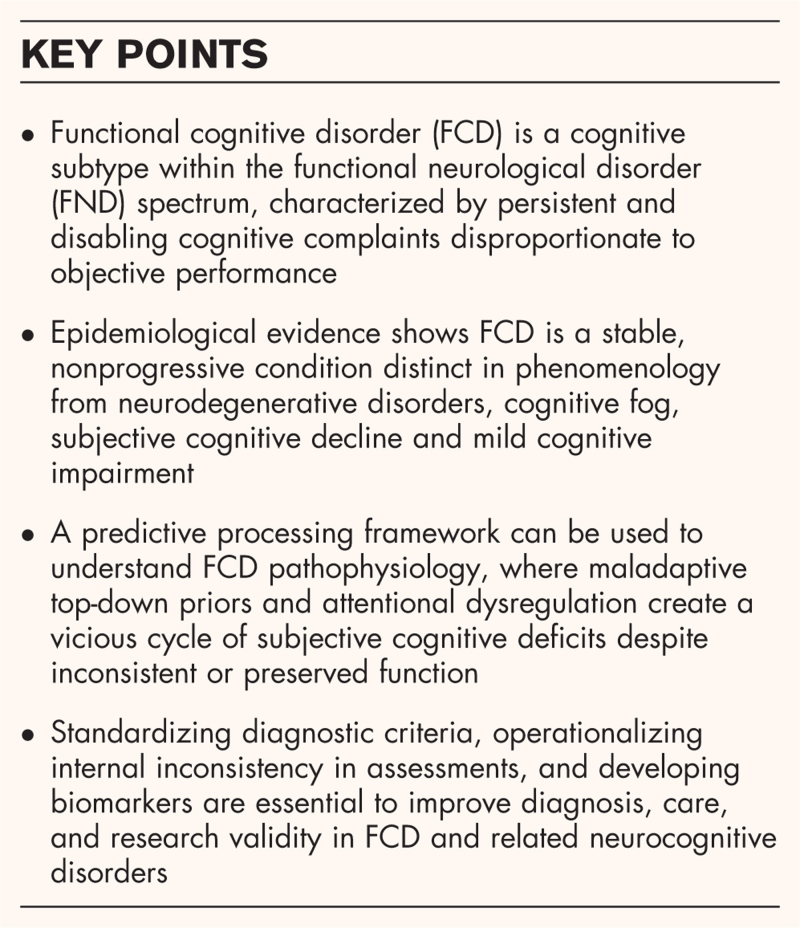
no caption available

## DEFINITIONS, EPIDEMIOLOGY AND NOSOLOGY – PROGRESS AND PITFALLS

Schmidtke *et al.* [16] first used the term “functional memory disorder” (FMD) and proposed a model that interpreted FMD as an acquired condition caused by psychological and emotional distress inducing chronic internal distractedness, leading to reduced ability to focus, maintain attention, and encode information. By comparison, the diagnostic approach for FCD is rooted in the current understanding of FND, emphasizing a “rule-in” diagnosis based on identifying specific, positive clinical features, rather than solely excluding other disorders or requiring distress as an essential criterion [17]. The core sign supporting an FCD diagnosis is internal inconsistency – a significant mismatch between severe subjective cognitive complaints and preserved abilities observed in conversation or testing – analogous to using Hoover's sign in establishing a motor FND diagnosis.

FCD conceptualization has been limited by varying terminology and diagnostic framing. In psychology clinics for instance, FCD symptoms are often framed as stress-related, or implied normal-age-related decline, invalidating for patients whose symptoms persist for years [13]. In psychiatry settings, individuals often receive nonspecific psychological disorder diagnoses resembling FCD (health anxiety, adjustment disorders, somatoform and anxiety disorders), or “depressive pseudodementia”.

The most robust argument supporting FCD however, is epidemiological. Studies indicate a prevalence of FCD ranging between 12% and 56% in new referrals seen in specialized memory clinics, often with affective symptoms and nonprogressive trajectories [18]. In a seminal FMD study by Schmidtke *et al.* [16], at 20-month follow-up, only 1 of 46 patients (2.1%) had progressed to dementia. When accurately diagnosed, FCD follows a stable, nonprogressive course [19] and diverges from the overlapping concepts of subjective cognitive decline (SCD), and mild cognitive impairment (MCI), both warranting discussion when juxtaposed against FCD. SCD serves as a broad descriptive term for cognitive complaints, and in research is framed as a prodrome to MCI and dementia [20] while MCI requires objective cognitive impairment without loss of independence. The SCD–MCI–dementia model contrasts with evidence that many people with subjective cognitive complaints do not progress to dementia, a discrepancy highlighted in literature [17] and emphasized in the earliest investigations of ‘presenile’ dementia [21]. While FCD patients can meet criteria for both SCD and MCI [22^▪▪^], FCD is differentiated by interactional differences representing heightened self-awareness of symptoms and detailed narratives of memory failures, contrasting with reduced insight (anosognosia) and vague symptom descriptions in neurodegenerative dementia [13]. The diagnostic challenge lies in recognizing that many MCI “nonprogressors” may represent undiagnosed FCD, with MCI sometimes serving as a “place-holder” due to insufficient clarity about FCD symptomatology [23].

Beyond its epidemiological distinctiveness, FCD is conceptualized as a functional disorder where cognitive symptoms are driven by dysfunction in metacognition, attentional dysregulation, and maladaptive cognitive-behavioural processes [24] such as, excessive self-monitoring, memory perfectionism, negative illness beliefs and fear conditioned avoidance of mental exertion (or cogniphobia) [15^▪▪^]. FCD has the hallmark feature of ‘internal inconsistency’ stemming from negatively biased appraisal of normal cognitive lapses where perceived subjective deficits are at odds with preserved or inconsistent objective performance [24].

The importance of inconsistency as the essential clinical feature of FCD is supported by a growing body of research [15^▪▪^,^18^] and aligns diagnostic practices with FND, where diagnosis is a rule-in process based on incongruence with neurological disorders or neuroanatomy [25]. In FCD, inconsistency encompasses variability in function and cognitive performance across patient and informant report, behaviour, psychological constructs and neuropsychological assessment, but challenges remain in applying these to individual diagnosis. Internal inconsistency in FCD remains poorly operationalized and subjective, with unclear delineation from cognitive variability in healthy individuals [12]. Eliciting internal inconsistency in clinical evaluations can vary among clinicians, leading to potential misdiagnosis, missed treatment and iatrogenic harm. Significant progress has been made validating clinical signs that demonstrate this feature across domains, contexts and time. Examples include characteristic communication profiles where patients provide detailed narratives of dense memory failures and extensive histories, paradoxically demonstrating preserved cognitive function. FCD patients for instance spoke for a median of 124-s when describing their memory concerns, markedly longer than the 42-s median in patients with neurodegenerative diagnoses [24]. Patients with FCD show conserved conversational fluency and preserved capacity to perform cognitively demanding occupations that contrast with their reported subjective complaints and have symptoms that fluctuate or are distractible. Behavioural features of inconsistency include patient concern being significantly higher than informants, evidenced by the “attending-alone-sign” or presentation of an organized written symptom list [12].

FCD characterized by subjective cognitive complaints that are disproportionate to objective performance, which places neuropsychological assessment at the centre of its identification. Despite this presumed role, relatively few neuropsychology publications have examined the cognitive profile of FCD or provided empirical demonstrations of inconsistency. Certain patterns on neuropsychological testing have been posited as potential indicators [26^▪▪^]. These include discordance between easier versus harder versions of related tasks, such as worse performance on repetition of digits forwards versus digits backwards, or worse performance on cued recall versus free recall. Relative to patients with neurodegenerative MCI, some FCD patients display normal delayed recall, with impaired immediate recall and recognition [27]. Notably in this study however, both patients with FCD and MCI performed poorly on a complex attention task (trail making), highlighting the problematic simplicity in defining inconsistency solely based on a subjective-objective mismatch. Neuropsychologists may seek out evidence of variable performance within cognitive domains, such as statistical discrepancy analysis of scatter between subtests [28], or inconsistency in performance at different time points though norms are lacking. Patients may demonstrate maladaptive strategies such as excessive emphasis on accuracy over speed or reluctance to approximate when uncertain [28]. FCD patients can also produce a profile that is incongruent with cognitive architecture, for example poor auditory attention span but intact encoding on verbal memory tasks. Metacognitive scales (i.e. the Multifactorial Memory Questionnaire, MMQ) [29] provide an estimate of perceived cognitive failures, but their futility is limited by paucity of base rate data and metacognitive discordance observed in healthy individuals, psychiatric illness and across different racial backgrounds [12]. Importantly, these inconsistencies within a neuropsychological evaluation are not unique to FCD. They occur in non-FCD presentations due to factors such as test anxiety, fatigue, or variable cognitive effort. Accordingly, inconsistent test patterns must be interpreted alongside characteristic testing behaviours and FCD-relevant themes in the clinical history and interview [30].

Finally, the use of performance validity tests (PVTs) as a diagnostic tool in FND remains controversial. High heterogeneity in failure rates ranging from 5% to 50% have been noted in FND [11], while a recent systematic review concluded that PVT failure was common across clinical conditions (including neurodegenerative disease and epilepsy) and that failure rates in FND were similar compared with other diverse neurological and psychiatric conditions [31]. While this finding has been criticized for oversimplifying the statistical and clinical context of PVT interpretation [32], its implications suggest that PVT failure alone cannot serve as confirmatory evidence of FCD, necessitating a broader clinical assessment. Further, this work to date has focused on cognitive PVTs and the potential role of symptom validity scales in FCD, such as patterns of somatic and cognitive symptom reporting on personality inventories, remains largely unexplored.

Taken together, while FCD remains difficult to define, phenotyping studies highlight key features distinguishing it from neurodegenerative and psychiatric disorders [17]. Several discriminative features have been formalized into screening instruments, like the Schmidtke Criteria [33] and an FCD diagnostic checklist [26^▪▪^], showing high discriminative accuracy against neurodegenerative conditions. While these represent laudable progress in the field, there remains a need to establish standardized evaluation processes [18]. A critical next step is to standardize the operationalization of inconsistency within neuropsychological assessment through FCD-specific test batteries that systematically probe for such discrepancies, and to establish clear guidelines for integrating patient-reported outcomes with objective findings.

## INTEGRATING THE EVIDENCE – APPLYING A PREDICTIVE PROCESSING FRAMEWORK TO FCD

While there is consensus on the mechanistic relevance of attentional and metacognitive dysfunction, agency deficits and maladaptive cognitive-behavioural processes in FCD [17], a unifying model linking symptom genesis and persistence with these factors would benefit patients, clinicians and neuroscientists alike and catalyse further research. Many models have been proposed, each adopting a particular thematic focus relevant to FCD. Some have emphasized a cognitive-behavioural framework where negative life events feed into cognitive distortions and attentional processes leading to symptom expression [12]. Others have connected triggering health events with dysfunctional cognitive schema and behavioural loops that underpin symptom persistence [15^▪▪^].

Comprehensive models have attempted to link computational neuroscience to neurobiological substrates in FND with Edwards *et al.* [34] first proposing a Bayesian brain model defining FND through predictive processing and active inference, with elaborations integrating emotional processing, interoception, agency and multinetwork dysfunction as key concepts tied to the FND phenotype [2,5]. Jungilligens and Perez [35^▪▪^] in their synthesis of constructed emotion theory integrated hierarchical prediction across exteroceptive (sensory-motor), interoceptive (bodily state), and metacognitive (self-representation) domains explaining how FND symptoms represented misattributed emotional arousal driven by dysregulated allostatic control and active inference. Many models however, extrapolate from motor-sensory FND to cognitive symptoms without addressing cognitive-specific features though recent years have seen metacognition explicitly embedded into explanatory frameworks offering a computational account of hierarchical metacognitive dissociation in FCD [36].

**FIGURE 1:**
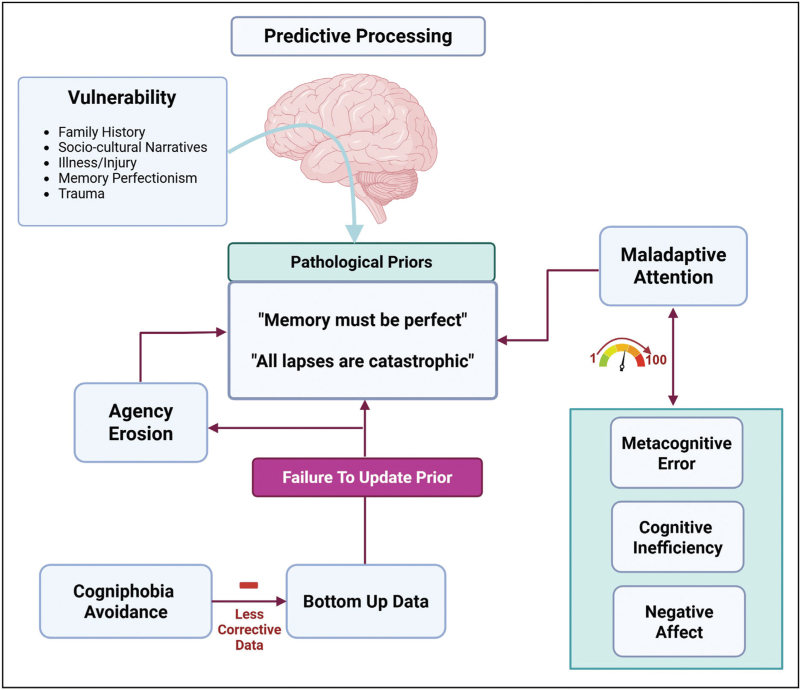
Predictive processing framework of functional cognitive disorder (FCD). This model illustrates how FCD arises from miscalibration within the brain's hierarchical Bayesian inferential system. Factors including acute stress, injury or illness, family history and cognitive-behavioural processes such as memory perfectionism serve to establish and reinforce threat-biased top-down priors (e.g., “my memory is failing”), which are overweighted relative to bottom-up sensory and performance feedback. A self-perpetuating cycle of error-prone predictions is established, maintaining subjective cognitive deficits despite preserved objective cognitive function while eroding agency and promoting metacognitive decoupling. Attentional dysregulation acts as a ‘gain modulator’, amplifying maladaptive self-monitoring and interacting bidirectionally with abnormal precision-weighting mechanisms (effortful cognition, negative affect and hyperarousal), further reinforcing pathological priors. Cogniphobia and task avoidance promote restricted sampling of contradictory evidence, further cementing dysfunctional priors resistant to disconfirmation thereby completing a vicious cycle of maladaptive predictive processing. Created in https://BioRender.com.

Although these models represent advances in understanding and validating FCD, cognitive symptoms present distinct challenges requiring refinement. Unlike motor symptoms (e.g., functional tremor) where abnormal sensory-motor prediction can be directly observed, cognitive symptoms involve higher-order predictions about internal mental operations and are inherently metacognitive (thinking about thinking). Evidence supports FCD pathophysiology as being grounded in predictive processing and active inference theory, where the brain operates within a hierarchical Bayesian inferential framework [37]. A putative failure of this system in accurately integrating abstract, context-dependent top-down priors with bottom up sensory and performance outcomes creates a self-perpetuating error-prone cycle that maintains FCD by eroding agency [38], learning and neural efficiency [39]. Attention acts as a ‘gain-modulator’ overweighting prior expectations while maladaptive attentional capture (hypervigilant self-monitoring, transformation of automatic to controlled processes, attention-dependent performance variability) serves as a dynamic driver of symptom variability and persistence whilst also providing an insight into the cognitive exhaustion, slowness and effortful thinking in FND patients [40]. Predisposing factors (illness, family history, acute stress, memory perfectionism [18]) help establish dysfunctional ‘priors’ reinforced by attentional dysregulation, negative affective states and catastrophic labelling of normal cognitive lapses rendering threat-biased priors resistant to disconfirmation [41]. Cogniphobia (fear and avoidance of cognitive exertion) is a behavioural outcome but also perpetuates restricted sampling of objective contradictory evidence of normality completing a vicious cycle of computational dysfunction (Fig. 1). Taken together, this framework suggests that FCD arises from a miscalibration of predictive models, where an overreliance on negatively biased priors (e.g., ’my memory is failing’) leads to an erroneous interpretation of normal cognitive fluctuations, creating a self-reinforcing cycle of perceived deficit despite preserved objective performance.

## CONCLUSION

There is a clear need to further refine FCD by standardizing norms that define internal inconsistency in operationalized, cognitive-specific terms. Establishing such criteria is essential to improving clinical care and research in FCD, ensuring more precise diagnosis and timely treatment. This refinement has the potential to advance our understanding of the pathophysiology of FND by clarifying mechanistic models and diagnostic boundaries. Furthermore, achieving greater trial cohort purity in neurodegenerative disease research and studies of disease-modifying therapies relies on this rigorous delineation, ultimately enhancing the validity of clinical trial outcomes. We argue that defining FCD is imperative to enhance diagnostic accuracy, optimize patient outcomes, and drive forward therapeutic innovation in both FCD and broader neurodegenerative disease contexts. Future research should focus on developing objective biomarkers or neuroimaging correlates of maladaptive predictive processing in FCD, and investigate whether interventions targeting these specific attentional and metacognitive biases can lead to improved clinical outcomes.

## Acknowledgements

*Thank you to Emily Swift and Fleur Harrison for help with manuscript preparation and to Gurpreet Kaur Hansra for assistance with the figure.*

### Financial support and sponsorship

*No direct funding for this work. The FND Clinic was supported by a grant from the Mindgardens Neuroscience Network, and funding from South Eastern Sydney Local Health District and the Australian Government Department of Health and Aged Care.*

### Conflicts of interest

*There are no conflicts of interest.*

## REFERENCES AND RECOMMENDED READING

Papers of particular interest, published within the annual period of review, have been highlighted as:

▪ of special interest
▪▪ of outstanding interest
